# Supplementary material for: Metallically gradated silicon nanowire and palladium nanoparticle composites as robust hydrogenation catalysts
Source: Commun Chem. 2020 Jun 26;3:81. doi: 10.1038/s42004-020-0332-z (PMC9814402; doi:10.1038/s42004-020-0332-z)
Supplement: Supplementary file 1 — Supplementary Information [file 42004_2020_332_MOESM1_ESM.pdf]

SUPPORTING INFOMATION

Metallically Gradated Silicon Nanowire and Palladium Nanoparticle Composites as Robust Hydrogenation Catalysts

Yoichi M. A. Yamada,[a]* Heeyoel Baek,[a] Takuma Sato,[a] Aiko Nakao,[b] and Yasuhiro Uozumi[c]

[a] RIKEN Center for Sustainable Resource Science, Wako, Saitama 351-0198, Japan

[b] Bioengineering Laboratory, RIKEN, Wako, dSaitama 351-0198, Japan

[c] Institute for Molecular Science (IMS), Okazaki, Aichi 444-8787, Japan

*ymayamada@riken.jp

1. Supplementary Methods

General experimental details

All reagents, purchased from TCI, Aldrich, Wako, MERCK, and Fluka, were used without further purification. Water was deionized with a Millipore system as a Milli-Q grade. NMR spectra were recorded with JEOL JNM-AL500 spectrometer (500 MHz) in $CDCl_3$ at 25 °C. For ^{1}H NMR spectra, proton chemical shifts (δ) are given in ppm relative to tetramethylsilane (0.00 ppm) in $CDCl_3$. Multiplicities are indicated by s (singlet), d (doublet), t (triplet), m (multiplet), and br (broad). For ^{13}C NMR spectra, carbon chemical shifts were internally referenced to the deuterated solvent signal of $CDCl_3$ (77.0 ppm).

TEM/EDX analysis was performed on a JEOL JEM-2100F/JED-2300T. SEM images were obtained on a JEOL JSM6330F. Inductively coupled plasma mass spectrometer (ICP-MS) was performed on a Perkin Elmer Nexion™ 300D.

The GC was measured by an Agilent 6850 with a capillary column (IL-60, 0.25 mm i.d. x 30 m or HP-1, 0.32 mm i.d. x 30 m). Mass spectrometric measurements were performed with an Agilent 7890B/ Agilent 75977B MSD equipped with a capillary column (HP-1ms, 0.25 mm i.d. x 30 m) and JMS-T100GCV (JEOL).

The XPS spectra were recorded by Thermo Scientific ESCALAB 250 with a monochromatic Al K_α X-ray source (1486.6 eV). The C s1 peak of contaminant carbon was calibrated to 284.8 eV. For XPS analysis, the sputtering was performed with an Ar ion gun at 3 keV. XAFS experiment using the synchrotron radiation was carried out at BL-13 of the SR Center in Ritsumeikan University (Shiga, Japan). Pd L_3-edge spectra were recorded with fluorescence X-ray yield (FLY) and total electron yield (TEY) method. FT-IR spectra were recorded by JASCO FT/IR-6200 in transmission mode for wafer

samples. UV-VIS absorption spectra were measured by JASCO V-650 at room temperature.

2-1. Preparation of a SiNA-Stabilized Pd Nanoparticle Catalyst

A boron doped p-type Si (100) wafer (0.1-100 Ω·cm; Φ = 2 inch; 1.5 g; S_{BET} 4 cm^2/ cm^2 (Kr)) was immersed in a mixture of conc. 95% H_2SO_4 and 30% H_2O_2 (15 mL : 5 mL, v/v) for 15 min., and then, washed with H_2O and dried with N_2 blow. The washed Si wafer was treated with 5% aqueous HF (10 mL) for 3 min, washed with H_2O, dried with N_2 blow. One side of Si wafer was masked with an urethane mask sheet (Kokuyo, Co. Ltd). Masked Si wafer was placed into a mixture of 46% HF (15 mL) and $AgNO_3$ (53.2 mg) in H_2O (47.6 mL), which was slowly stirred for 1min. The Ag –coated Si wafer was washed with H_2O, and dried with N_2 blow. The urethane mask sheet was peeled off. The Ag-coated Si wafer was placed into a mixture of 46%HF (4.0 mL) and 30% H_2O_2 (0.9 mL) in H_2O (19.0 mL) at 60 °C for 3 min, and Si wafer was washed with H_2O and dried with N_2 blow. The etched Si wafer was immersed twice in 50% aqueous HNO_3 (30 mL) for 3 min, washed with H_2O, dried with N_2 blow to give the Si nanowire array (SiNA). After SiNA was placed into 5% aqueous HF (10 mL) for 1 min, washed with H_2O, dried with N_2 blow, it was immersed in a mixture of 50 mM aqueous K_2PdCl_4 (4.5 mL) and acetone (1.5 mL) for 5 min. The Si wafer was washed with H_2O and acetone, and dried with N_2 blow to give SiNA-stabilized Pd nanoparticle catalyst (SiNA-Pd). The loading of Pd was determined by ICP-MS.

2-2. General Procedure for the Hydrogenation of Various Substrates

Table 1.

To a 10 mL glass vessel was added SiNA-Pd (0.00025 mmol, 0.05 mol% Pd), each substrate (0.5 mmol), and ethanol (2 mL). The reaction was performed with H_2 (0.1 MPa, balloon) at 70 °C for 12-24 h. After cooling to room temperature, the reaction mixture was analyzed with GC (HP-1) with dodecane or mesitylene as an internal standard to determine the yield.

GC condition of product **2d**, **2e**, and **2f:**
GC: Agilent 6850 with a capillary column.
Column: HP-1, 0.32 mm i.d. x 30 m.
Injection volume: 1μL.
Injection mode: Splitless.
Inlet temperature: 250 °C.
Oven: 50 °C (3 min), 20 °C/min to 300 °C (10 min).
Carrier gas: He

GC condition of product **2h:**
GC: Agilent 6850 with a capillary column.
Column: HP-1, 0.32 mm i.d. x 30 m.
Injection volume: 1μL.
Injection mode: Splitless.
Inlet temperature: 250 °C.
Oven: 35 °C (3 min), 20 °C/min to 300 °C (10 min).
Detector: FID, 300 °C.
Carrier gas: He

- Oleic acid

To a 10 mL glass vessel was added SiNA-Pd (0.00025 mmol, 0.05 mol% Pd) and oleic acid (0.5 mmol). The reaction was performed with H_2 (0.1 MPa, balloon) at 70 °C for 24 h under the vortex mixing conditions by a vortex mixer with a temperature controller. After cooling to room temperature, the reaction mixture was analyzed by ^{1}H NMR and the yield was determined without further purification. The catalyst was recovered by picking up with a tweezer and washed with EtOAc/ Acetone/Water. After drying with N_2 blow, the catalyst was used for the next reaction.

- Linoleic acid and triolein

To an autoclave reactor was added SiNA-Pd (0.0006 mmol, 0.12 mol% Pd) and each substrate (0.5 mmol). The reaction was performed with H_2 (0.6 MPa) at 120 °C for 18 or 30 h. After cooling to room temperature, the reaction mixture was analyzed by ^{1}H NMR and the yield was determined without further purification. The catalyst was recovered by picking up with a tweezer and washed with EtOAc/Acetone/Water. After drying with N_2 blow, the catalyst was used for the next reaction.

2-3. Catalyst Reuse Experiments

To a 10 mL glass vessel was added SiNA-Pd (0.00025 mmol, 0.05 mol% Pd), *trans*-Stilbene (0.5 mmol, 90 mg), ethanol (2 mL), and dodecane as an internal standard. The reaction was performed with H_2 (0.1 MPa, balloon) at 70 °C for 24 h under the vortex mixing conditions by a vortex mixer with a temperature controller. After cooling to room temperature, the reaction mixture was analyzed with GC (HP-1) to determine the yield. The catalyst was recovered by picking up with a tweezer and washed with EtOAc/ Acetone/Water. After drying with N_2 blow, the catalyst was used for the next reaction.

2-4. ICP-MS Analysis

A. Metal Loading of SiNA-Pd

Determination of Pd loading was performed with the 1.42 Ultrapure HNO_3 (7 mL) and decomposed by the microwave assisted equipment. And then, the decomposed solution was analyzed by ICP-MS for the palladium content.

-results: 14.0 μmol/g or 13.54 μmol/g or 8.43 μmol/g.

B. Leaching Test of SiNA-Pd

A leaching test for SiNA-Pd was performed with the reaction mixture in hydrogenation of trans-stilbene. After the 127 run of catalytic hydrogenation, the reaction mixture was filtered through 0.2 μm PTFE syringe filter, evaporated, and dried under vacuum. And then, the filtrate (43.2 mg) was put into the 1.42 Ultrapure HNO_3 (7 mL) and decomposed by the microwave assisted equipment. The decomposed solution was analyzed by ICP-MS for the palladium content.

- *result: less than limit of detection* (*limit of detection: 0.021 ppb*).

2-5. IR experiment

The infrared (IR) absorption spectra of SiNS-Pd and SiNS-H in transmission mode (Supplementary Figure 1) indicate that the hydro-gen-terminated silicon surface was oxidized during the treatment with Pd(II). Thus, the IR spectra of the wafers suggest that the surface silicon hydrides (Si-H_x, x = 1, 2, 3) were completely consumed during the Pd-treatment (Supplementary Figure 1a). The second derivative of the spectrum of SiNS-H (Supplementary Figure 1b) revealed the surface Si-H_x stretching vibrations (denoted as features a-e), where the absorptions were assigned as follows: the

asymmetric stretching vibration of coupled Si-H (a, 2070 cm^{-1}), symmetric stretching of coupled Si-H (b, 2089 cm^{-1}), asymmetric stretching of Si-H_2 (c, 2100 cm^{-1}), symmetric stretching of Si-H_2 (d, 2114 cm^{-1}), and Si-H_3 (e, 2136 cm^{-1}).[3,4,5] No formation of amorphous silicon hydride (a-Si:H, typically observed near 2000 cm^{-1}) was observed in the spectrum of SiNS-H. Meanwhile, both silicon oxide layer (Supplementary Figure 1c) and surface silanol group (Supplementary Figure 1d) were generated after the Pd-treatment. In addition, for each Pd(II) solution phase before and after the Pd-treatment, no significant change was observed by UV-VIS absorption (**Supplementary Figure** 2). This observation also suggests that the reduction of Pd(II) to Pd(0) did not occur in solution phase but at the interface between Pd(II) solution and the hydrogen-terminated silicon surface.

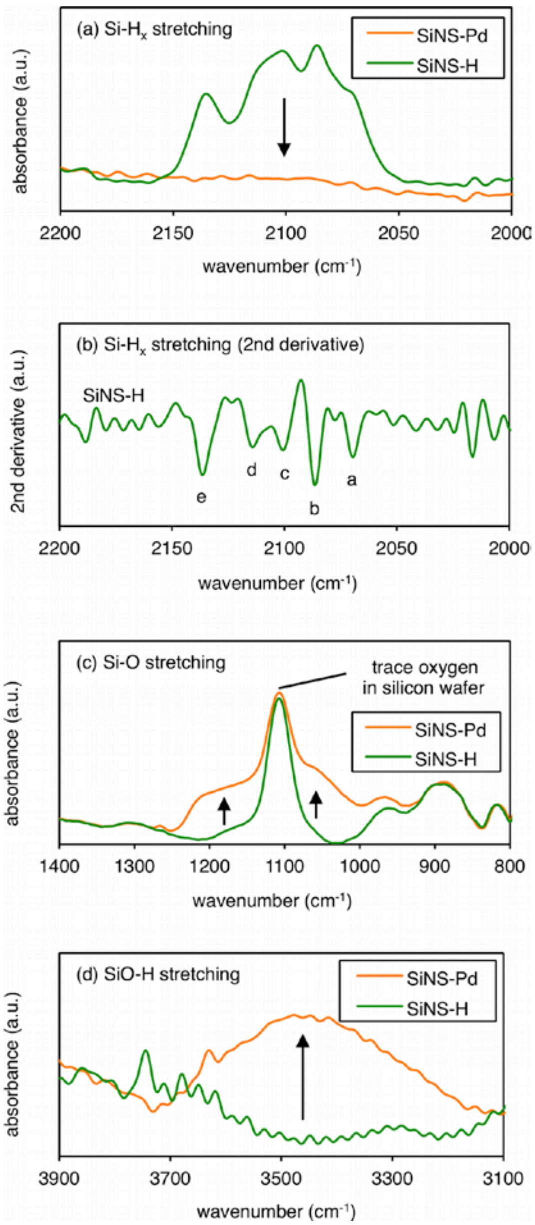

Supplementary Figure 1. Transmission IR spectra of SiNS-Pd and SiNS-H; (a): Si-H_x stretching, (b): the second derivative of (a) (shown only for SiNS-Pd), (c): Si-O stretching, and (d): SiO-H stretching vibrations.

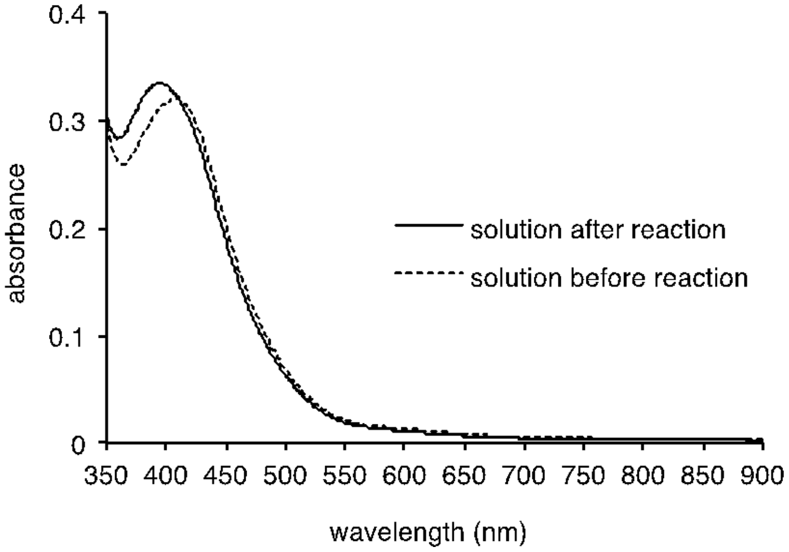

Supplementary Figure 2. UV-VIS spectra of solution phase before and after palladium nanoparticle formation on the nanostructured silicon surface.

2-6. SEM and TEM images

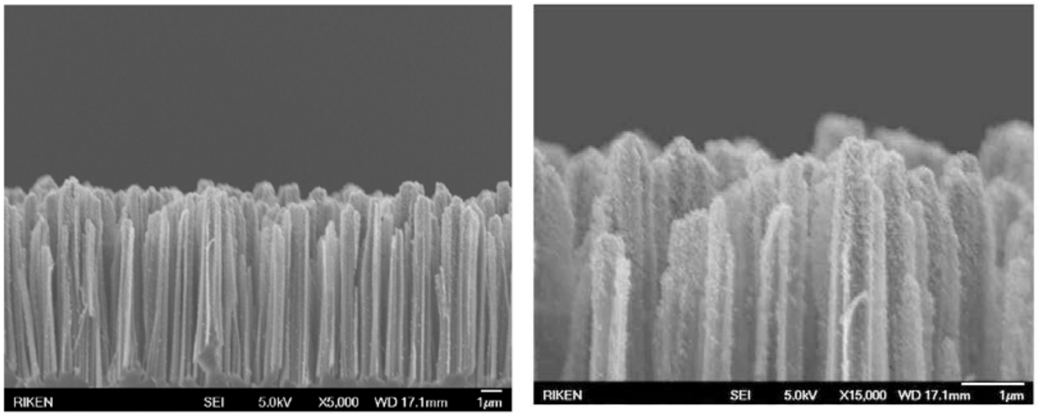

Supplementary Figure 3. SEM of SiNA-Pd

.

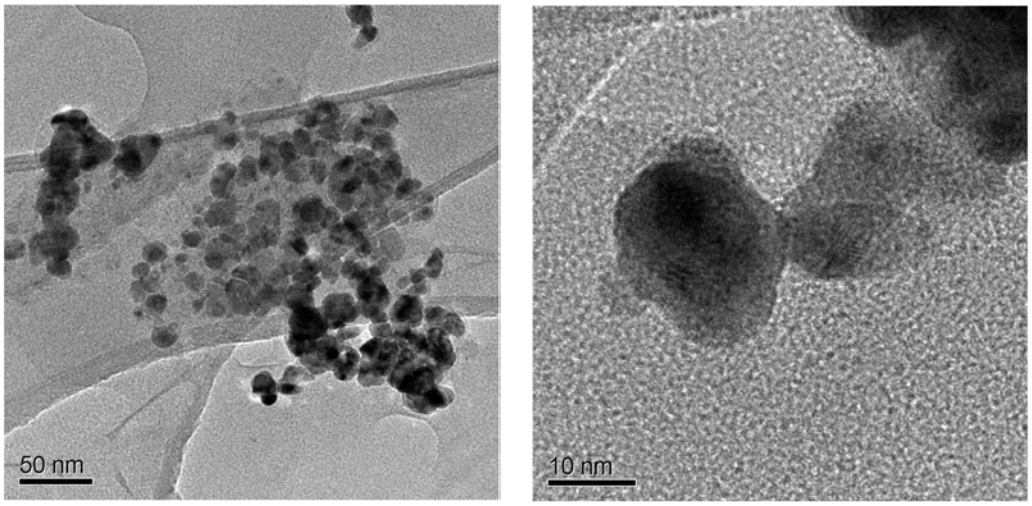

Supplementary Figure 4. High resolution TEM of Pd nanoparticles.

2-7. Characterization of the Compounds

^{1}H, ^{13}C NMR and GC of Products

(Z)-But-2-ene-2,3-diyldibenzene (1b)

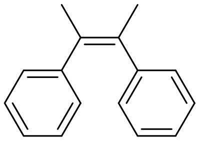

Zn dust (6-9 μm, 17.3g, 265 mmol) was suspended in THF (100 mL) under N_2. $TiCl_4$ (25 g, 132 mmol) was added to the resulting mixture, and the mixture was heated to reflux for 40 min to form dark purple solution. Acetophenone (5.1 mL, 44 mmol) was added to the resulting mixture at room temperature and heated to reflux overnight. The reaction mixture was quenched with H_2O (100 mL), and filtered by a paper filter to remove insoluble inorganic materials. The organic material was extracted with EtOAc (200 mL), washed with brine (50 mL), and dried over Na_2SO_4. After evaporation, the residue was purified by silica gel using hexane as an eluent to obtain the product in

70% yield (3.20 g) as a mixture of isomers. The product was further purified by recrystallization from EtOH to obtain analytically pure (*Z*)-2,3-diphenylbut-2-ene in 39% yield (1.78 g).

^{1}H NMR (500 MHz, $CDCl_3$): δ 2.16 (s, 6H), 6.95-6.96 (m, 4H), 7.00-7.03 (m, 4H), 7.06-7.09 (m, 2H) ; ^{13}C NMR (125 MHz, $CDCl_3$): δ 21.46, 125.50, 127.51, 129.16, 132.87, 144.65; MS (EI): *m/z* 208.1 $[M]^+$.

(3-Methylbut-2-en-2-yl)benzene (1c)[2]

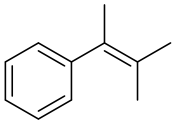

MeMgCl in THF (250 mL, 1 M, 250 mmol) was dropwise added to a solution of isobutyrophenon (31.5 mL, 210 mmol) in THF (200 mL) at 0 °C under N_2. After completion of addition, the reaction mixture was warmed to room temperature and reacted overnight. The reaction mixture was quenched by saturated NH_4Cl solution (200 mL) at 0 °C and diluted with H_2O (200 mL). The crude material was extracted with Et_2O (200 mL), washed with brine, and dried over Na_2SO_4. After removal of volatile materials under reduced pressure, 3-methyl-2-phenylbutan-2-ol was obtained in nearly quantitative yield. The resulting material was used for the following synthesis of 2-methyl-3-phenylbut-2-ene without further purification.

Concentrated H_2SO_4 (2 mL) was dropwise added to a solution of 3-methyl-2-phenylbutan-2-ol (3.28 g, 20 mmol) in AcOH (15 mL) at 70 °C, and the mixture was reacted for 10 min at 70 °C. The reaction mixture was poured into cold H_2O, and the crude material was extracted with pentane (2 × 100 mL), washed with saturated $NaHCO_3$ solution, and dried over Na_2SO_4. After concentration under mild reduced pressure (680 hPa, 35 °C), the residue was passed through a short pad of silica

gel using pentane as an eluent. After removal of solvent (420 hPa, 40 °C), 2-methyl-3-phenylbut-2-ene was obtained in 96% yield (2.80 g).

^{1}H NMR (500 MHz, $CDCl_3$): δ 1.59 (s, 3H), 1.81 (s, 3H), 1.96 (s, 3H), 6.95-6.96 (m, 4H), 7.00-7.03 (m, 4H), 7.06-7.09 (m, 2H); ^{13}C NMR (125 MHz, $CDCl_3$): δ 20.53, 20.78, 22.04, 125.68, 127.18, 127.90, 128.40, 129.98, 145.44; MS (EI): *m/z* 146.1 $[M]^+$.

1,2-Diphenylethane (2a)

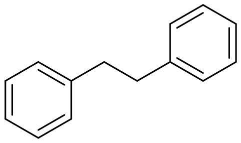

^{1}H NMR (500 MHz, $CDCl_3$): δ 1.54 (s, 4H), 7.18-7.21 (m, 4H), 7.27-7.30 (m, 6H); ^{13}C NMR (125 MHz, $CDCl_3$): δ 37.93, 125.89, 128.32, 128.43, 141.78; MS (EI): *m/z* 182.1$[M]^+$.

Butane-2,3-diyldibenzene (2b)

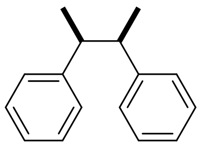

^{1}H NMR (500 MHz, $CDCl_3$): δ 1.01-1.03 (m, 6H), 2.76-2.83 (m, 2H), 7.20-7.23 (m, 6H), 7.30-7.33 (m, 4H); ^{13}C NMR (125 MHz, $CDCl_3$): δ 21.04, 47.26, 126.05, 127.62, 128.28, 146.50; MS (EI): *m/z* 210.1$[M]^+$.

(3-Methylbutan-2-yl)benzene (2c)

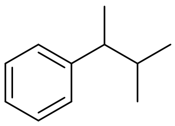

^{1}H NMR (500 MHz, $CDCl_3$): δ 0.74-0.76 (d, J=6.5 Hz, 3H), 0.92-0.94 (d, J=6.5 Hz, 3H), 1.22-1.24

(d, *J*=7.5 Hz, 3H), 1.72-1.81 (m, 1H), 2.40-2.44 (m, 1H), 7.14-7.18 (m, 3H), 7.27-7.29 (m, 2H); ^{13}C NMR (125 MHz, $CDCl_3$): δ 18.74, 20.16, 21.16, 34.42, 46.85, 125.65, 127.64, 128.00, 147.09; MS (EI): *m/z* 148.1[M]$^+$.

Octadecane (2g)

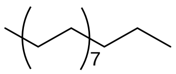

^{1}H NMR (500 MHz, $CDCl_3$): δ 0.87-0.89 (t, *J*=6.5 Hz, 6H), 1.25-1.33 (m, 32H); ^{13}C NMR (125 MHz, $CDCl_3$): δ 14.13, 22.70, 29.37, 29.66, 29.71, 31.93; MS (EI): *m/z* 254.3 [M]$^+$.

N-benzylaniline (2i)

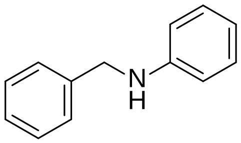

^{1}H NMR (500 MHz, $CDCl_3$): δ 4.03 (br s, 1H), 4.33 (s, 2H), 6.63-6.65 (m, 2H), 6.70-6.73 (m, 1H), 7.15-7.20 (m, 2H), 7.26-7.29 (m, 1H), 7.33-7.39 (m, 4H); ^{13}C NMR (125 MHz, $CDCl_3$): δ 48.31, 112.80, 117.53, 127.18, 127.47, 128.59, 129.21, 139.38, 148.09; MS (EI): *m/z* 183.1 [M]$^+$.

Stearic acid (2j)

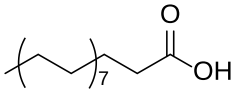

^{1}H NMR (500 MHz, $CDCl_3$): δ 0.87-0.89 (t, *J*=6.5 Hz, 3H), 1.25-1.3 (m, 28 H), 1.60-1.66 (m, 2H), 2.34-2.36 (t, *J*=7.5 Hz, 2 H); ^{13}C NMR (125 MHz, $CDCl_3$): δ 14.13, 22.70, 24.70, 29.07, 29.25, 29.37, 29.45, 29.60, 29.65, 29.66, 29.70, 31.93, 33.88, 179.03; MS (EI): *m/z* 298.3 [M]$^+$.

Propane-1,2,3-triyl tristearate (2k)

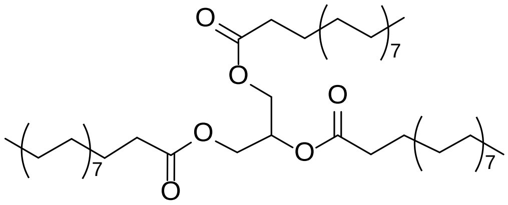

^{1}H NMR (500 MHz, $CDCl_3$): δ 0.87-0.89 (t, *J*=6.5 Hz, 9H), 1.25-1.31 (m, 84H), 1.59-1.62 (m, 6H), 2.30-2.33 (m, 6H), 4.13-4.16 (m, 2H), 4.28-4.31 (dd, J=12 Hz, 4.5 Hz, 2H), 5.25-5.29 (m, 1H); ^{13}C NMR (125 MHz, $CDCl_3$): δ 14.1, 22.7, 24.9, 29.1, 29.1, 29.3, 29.4, 29.5, 29.6, 29.7, 29.7, 31.9, 34.1, 62.1, 68.9, 173.3; MS (ESI): *m/z* 913.8 [M+Na].

3. Supplementary References

1. M. C. Burla, R. Caliandro, M. Camalli, B. Carrozzini, G. L. Cascarano, L. De Caro, C. Giacovazzo, G. Polidori, R. Spagna, *J. Appl. Cryst.* **2005**, *38*, 381-388.

2. G. M. Sheldrick, A*cta Crystallogr. Sect. C*, **2015**, *C71*, 3-8.

3. G. Lucovsky, *J. Non-Cryst. Solids* **1992**, *141*, 241-256.

4. Y. J. Chabal, *Surf. Sci.* **1986**, *168*, 594-608.

5. P. Jakob, Y. J. Chabal, K. Kuhnke, S. B. Christman, S*urf. Sci.* **1994**, *302*, 49-56.

4. Supplementary Figure

GC and NMR chart

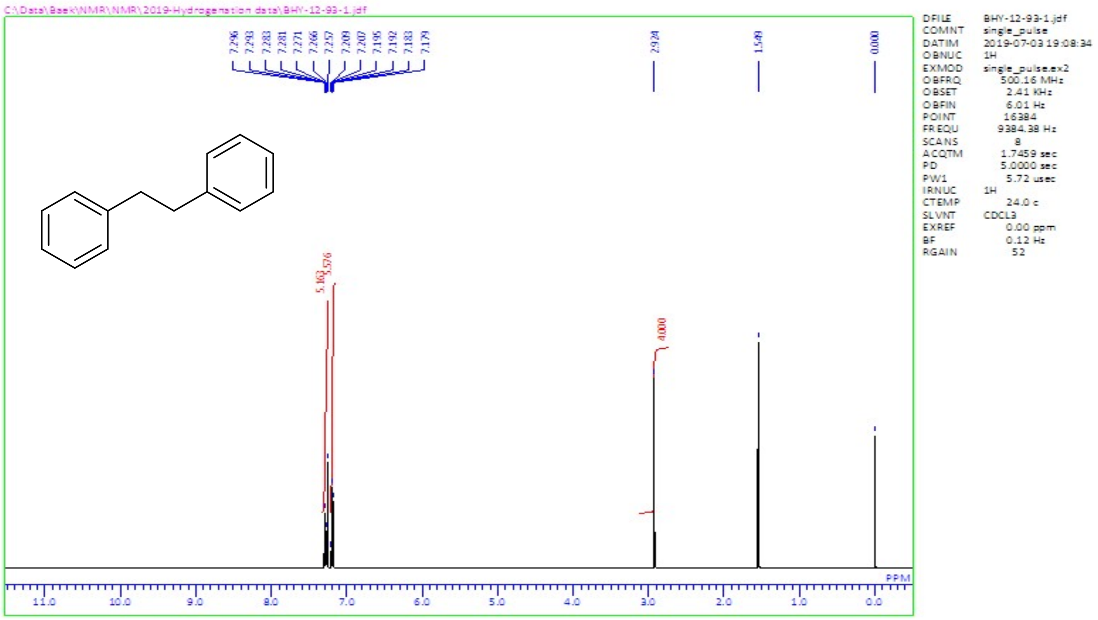

Supplementary Figure 5. ^{1}H NMR Spectra of product **2a**

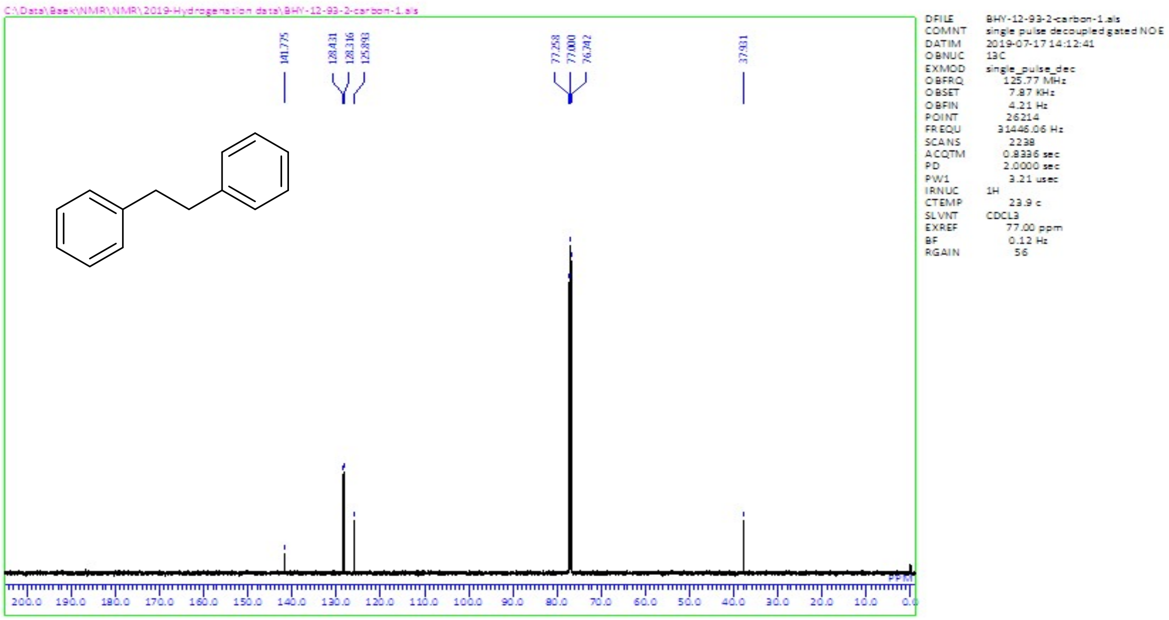

Supplementary Figure 6. ^{13}C NMR Spectra of product **2a**

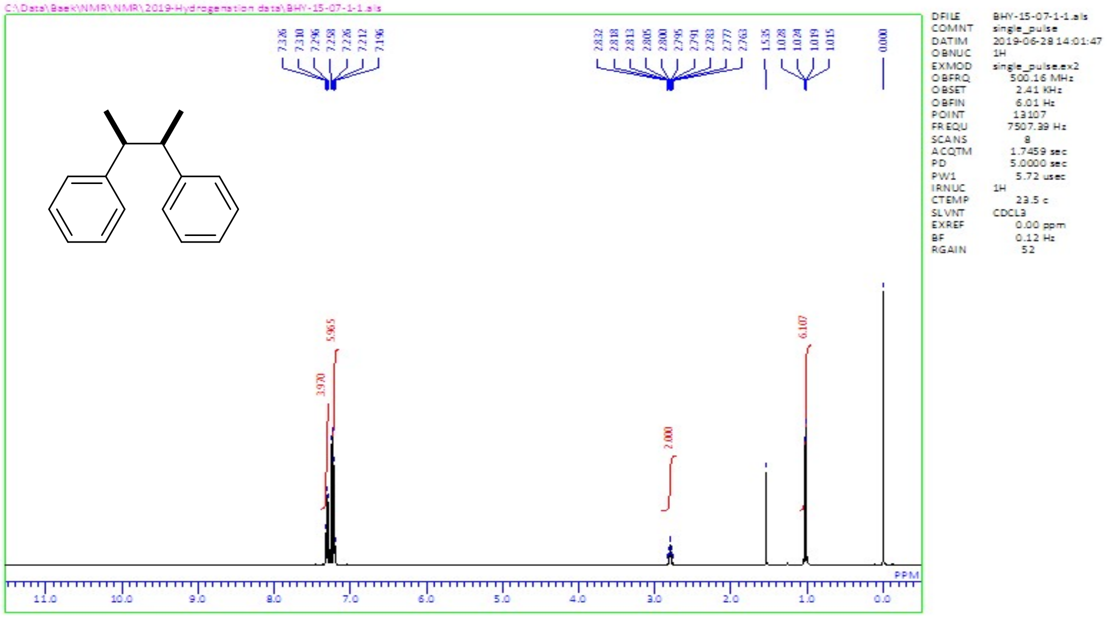

Supplementary Figure 7. ^{1}H NMR Spectra of product **2b**

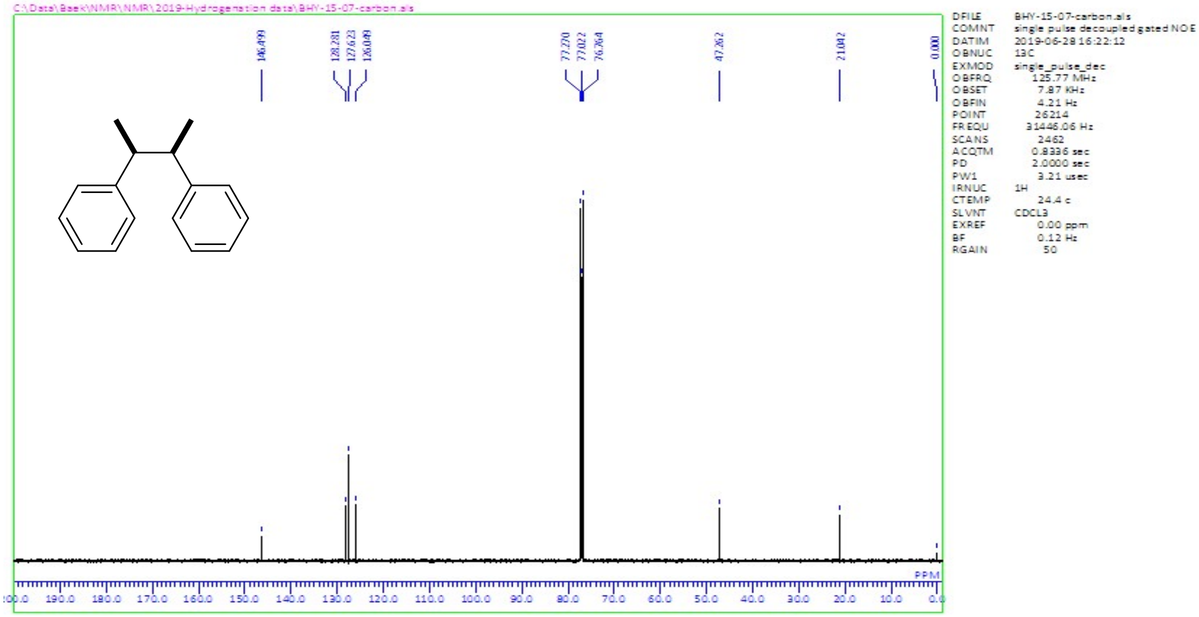

Supplementary Figure 8. ^{13}C NMR Spectra of product **2b**

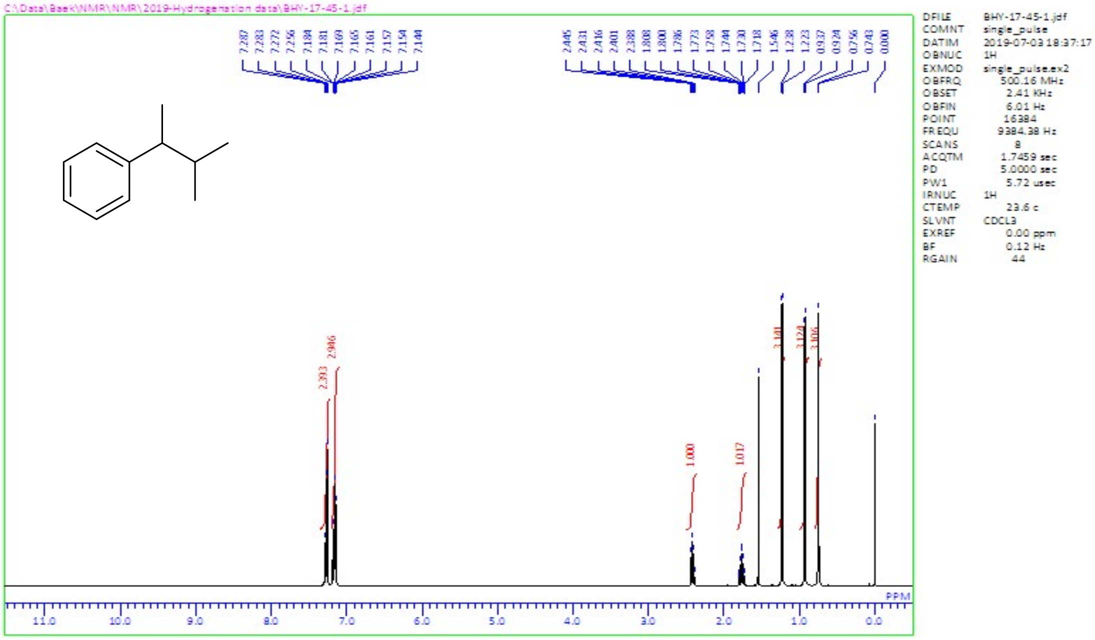

Supplementary Figure 9. ^{1}H NMR Spectra of product **2c**

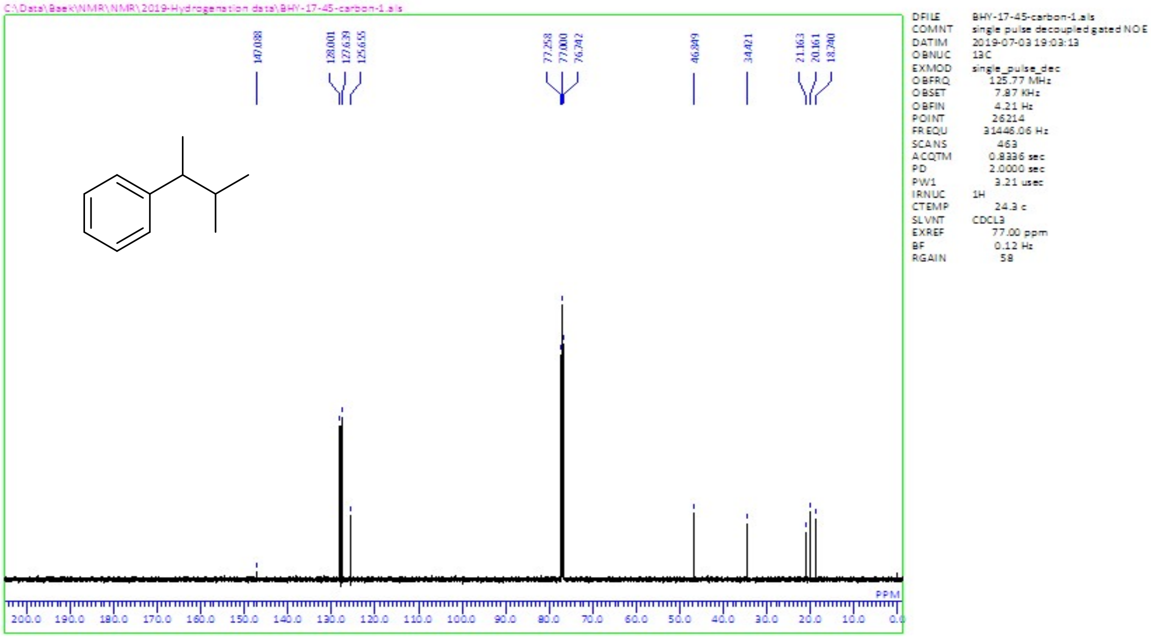

Supplementary Figure 10. ^{13}C NMR Spectra of product **2c**

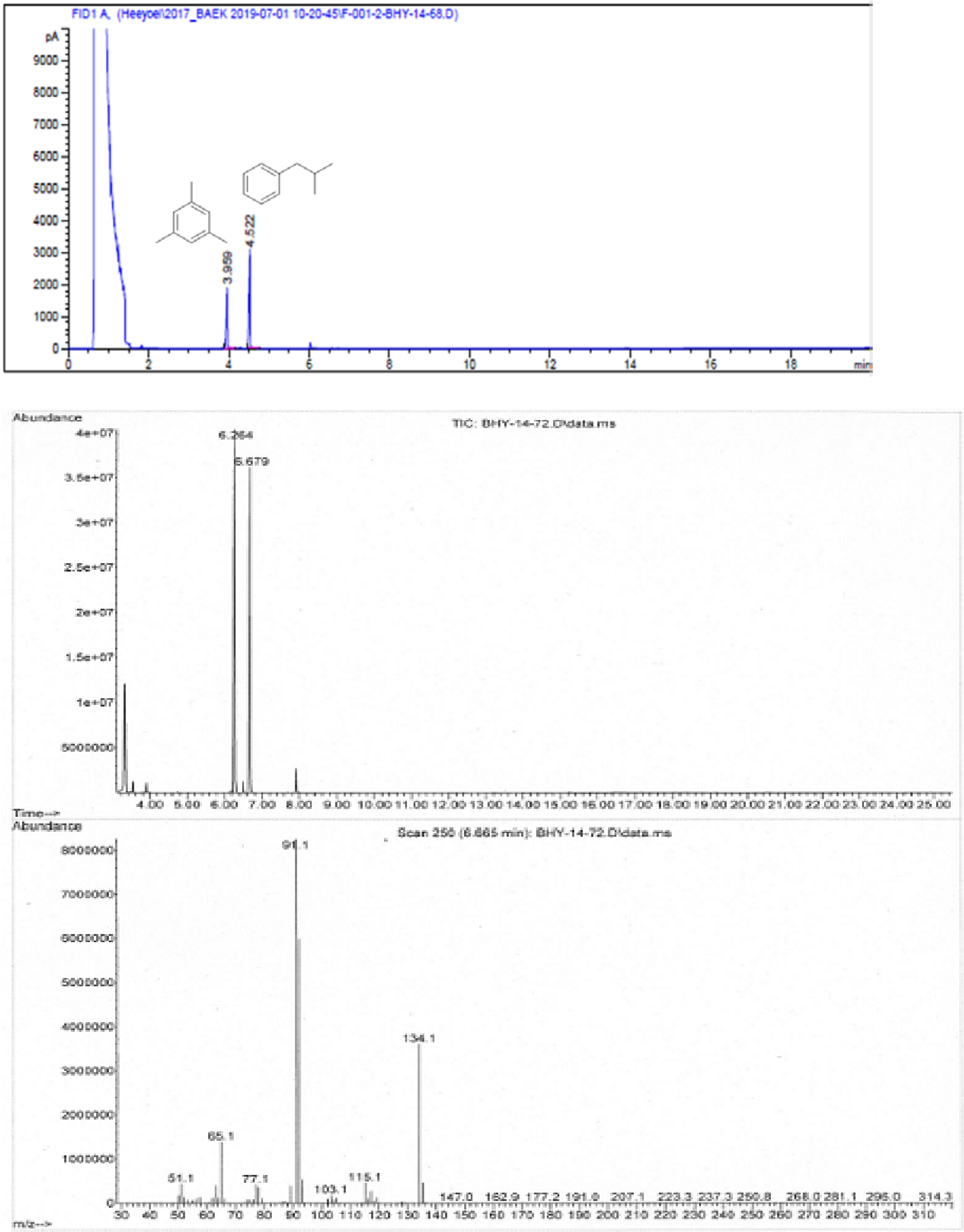

Supplementary Figure 11. GC and GC-MS spectra of product **2d**

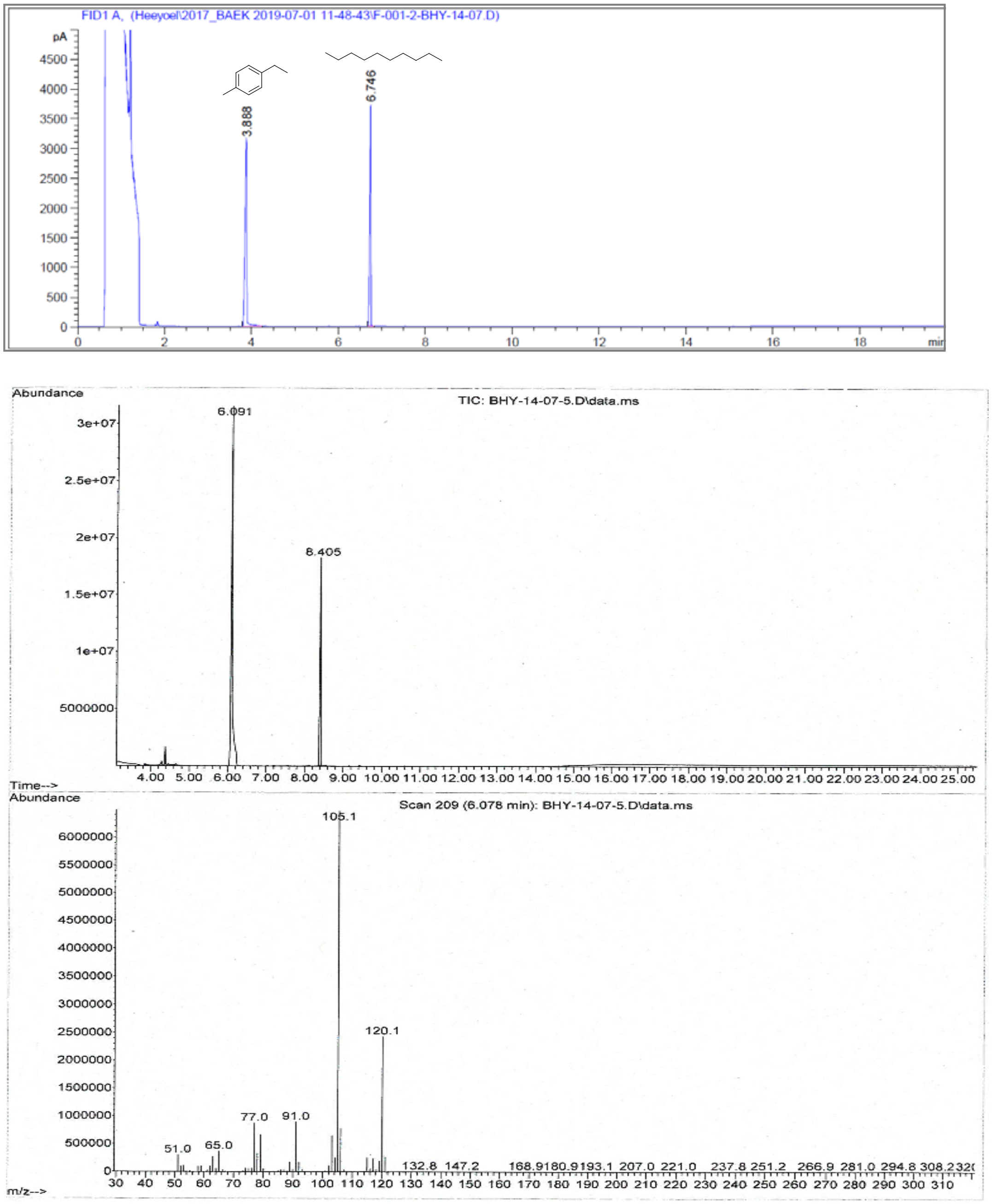

Supplementary Figure 12. GC and GC-MS spectra of product **2e**

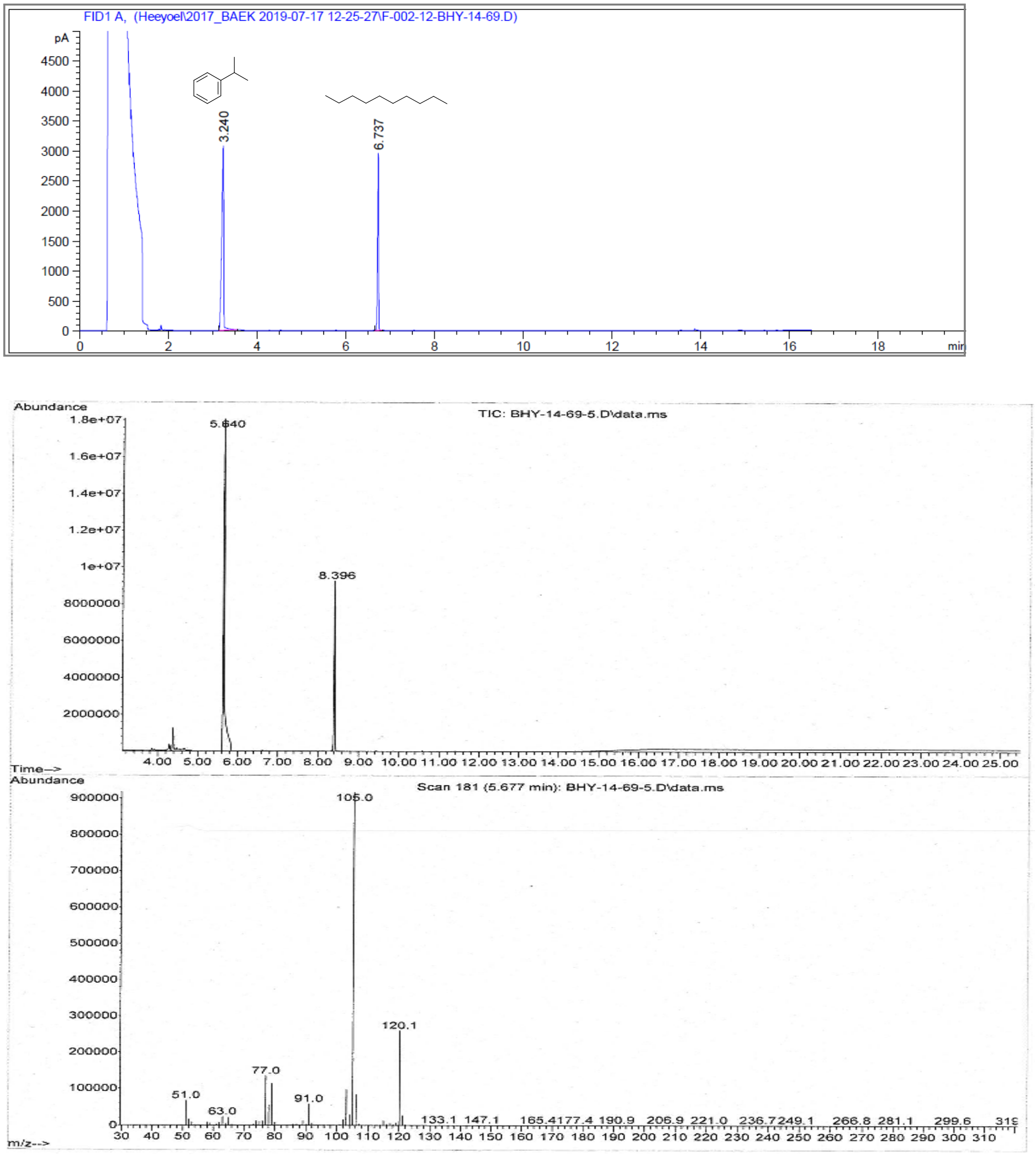

Supplementary Figure 13. GC and GC-MS spectra of product **2f**

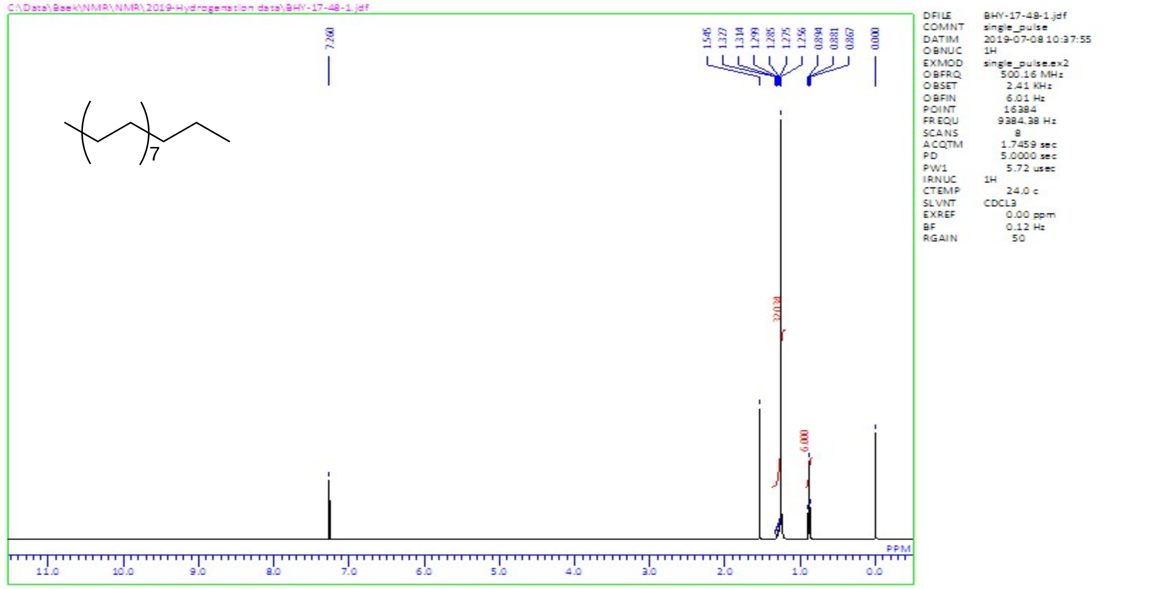

Supplementary Figure 14. ^{1}H NMR Spectra of product **2g**

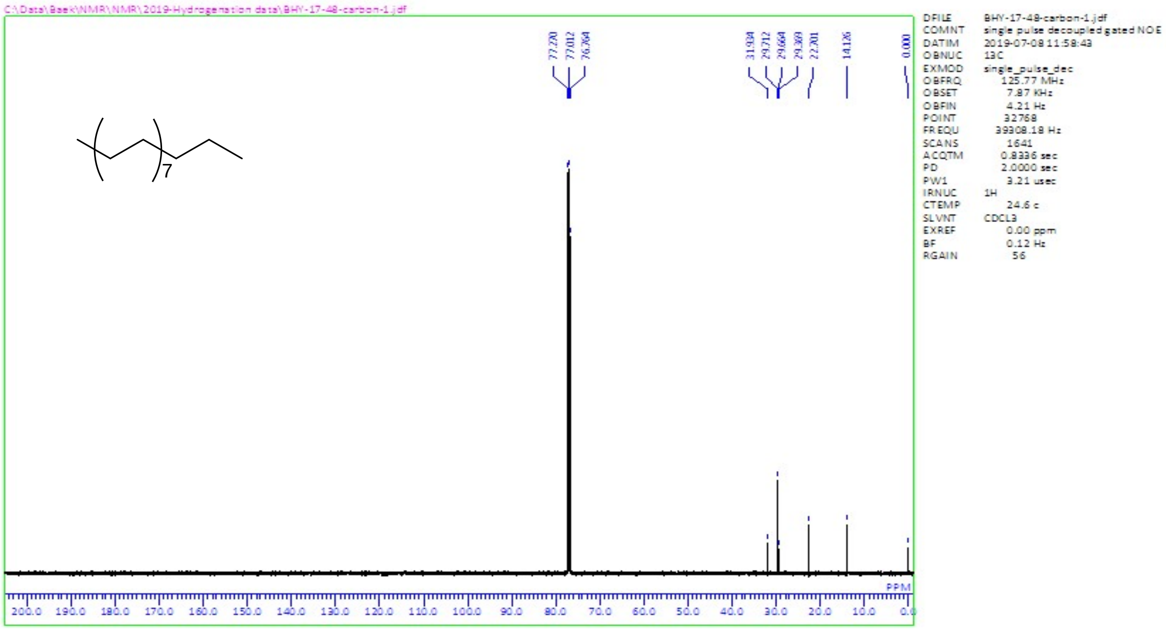

Supplementary Figure 15. ^{13}C NMR Spectra of product **2g**

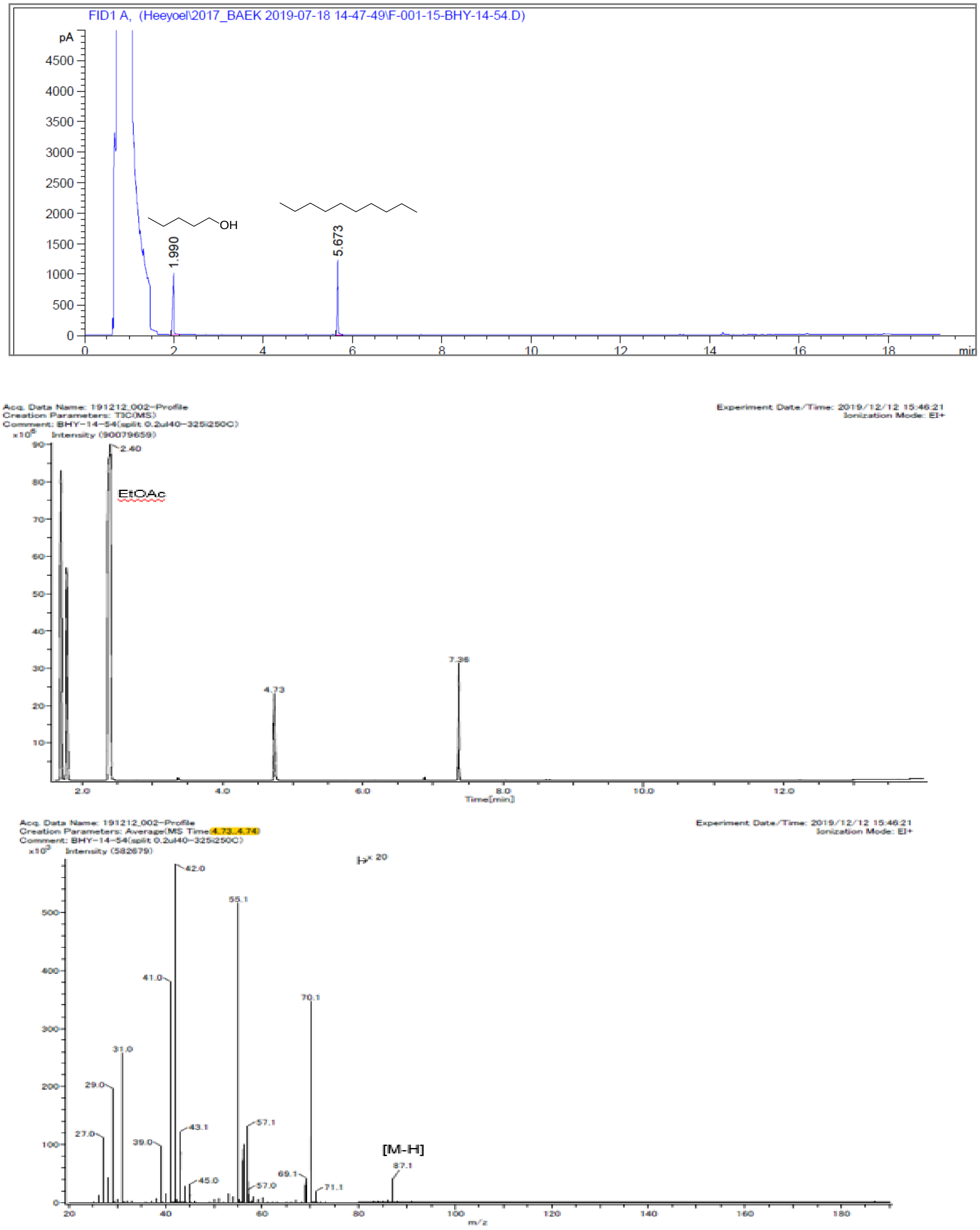

Supplementary Figure 16. GC and GC-MS spectra of product **2h**

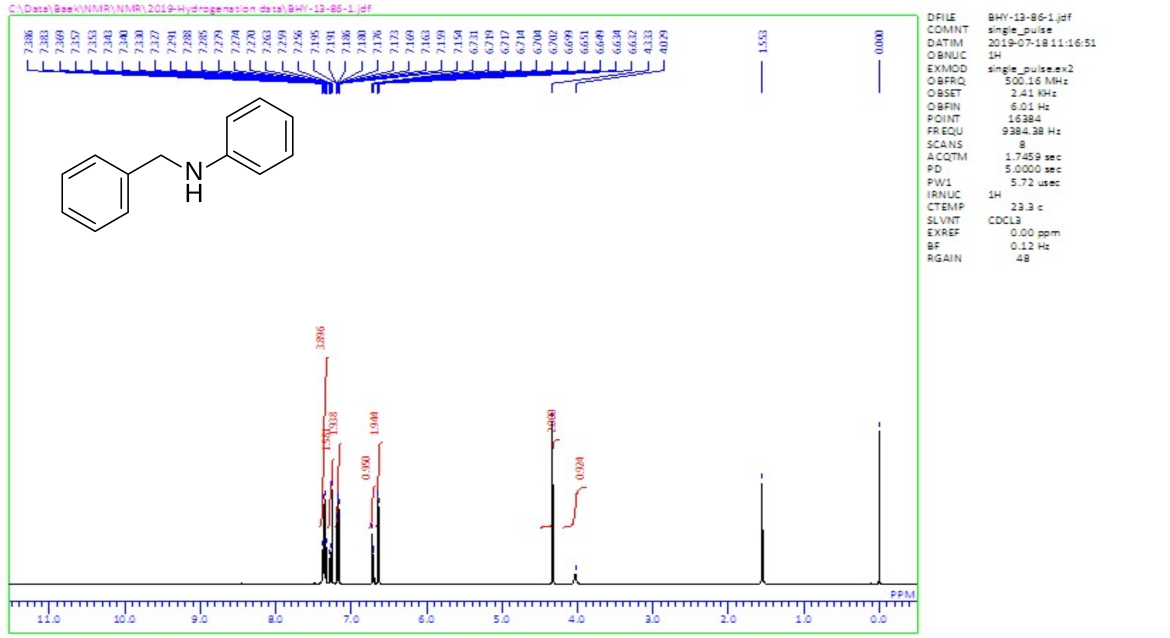

Supplementary Figure 17. ^{1}H NMR Spectra of product **2i**

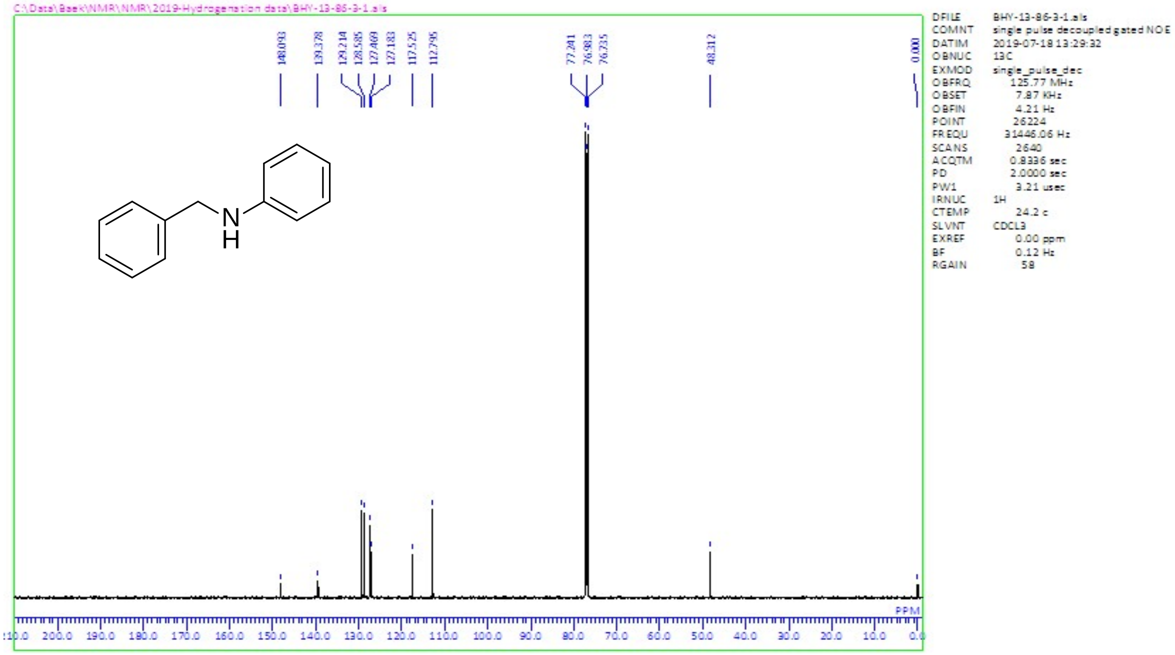

Supplementary Figure 18. ^{13}C NMR Spectra of product **2i**

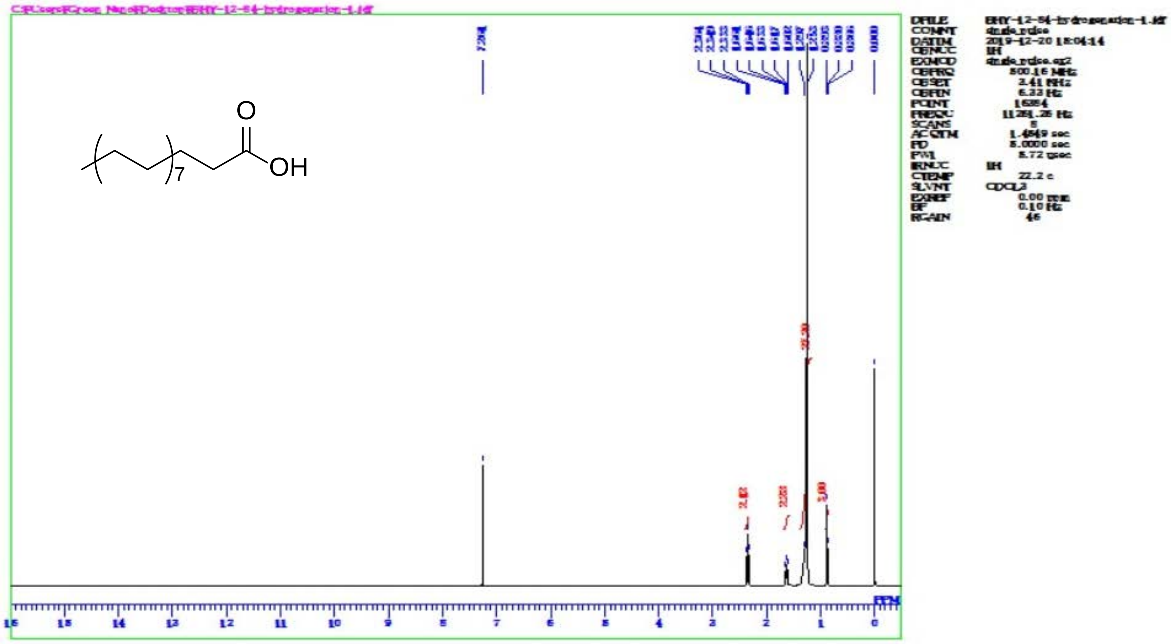

Supplementary Figure 19. ^{1}H NMR Spectra of product **2j**

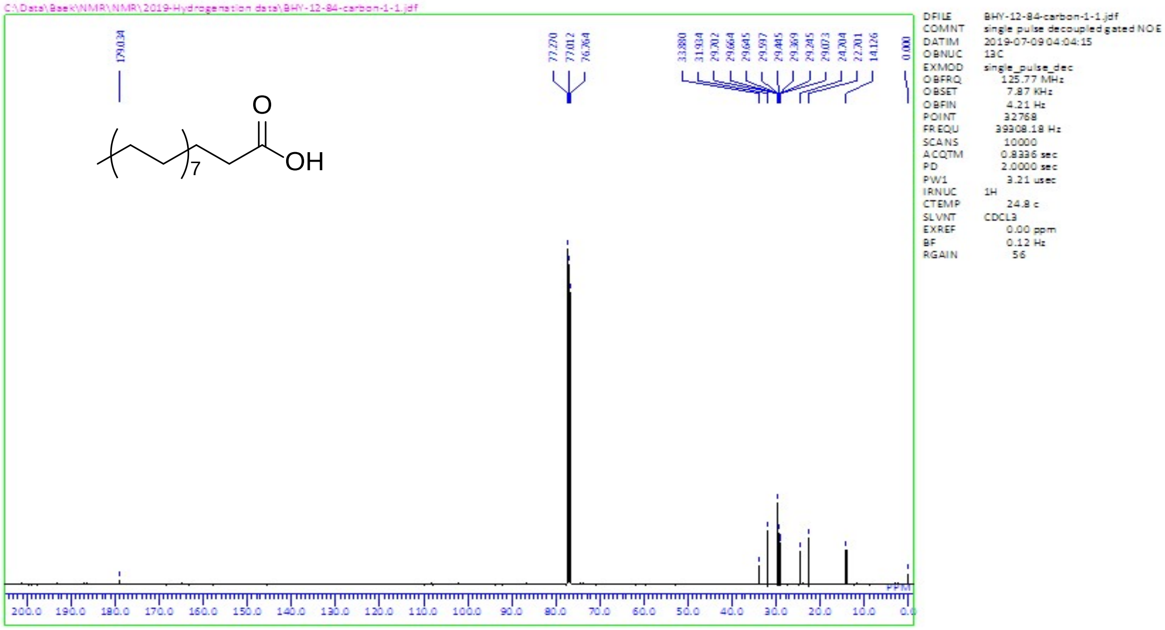

Supplementary Figure 20. ^{13}C NMR Spectra of product **2j**

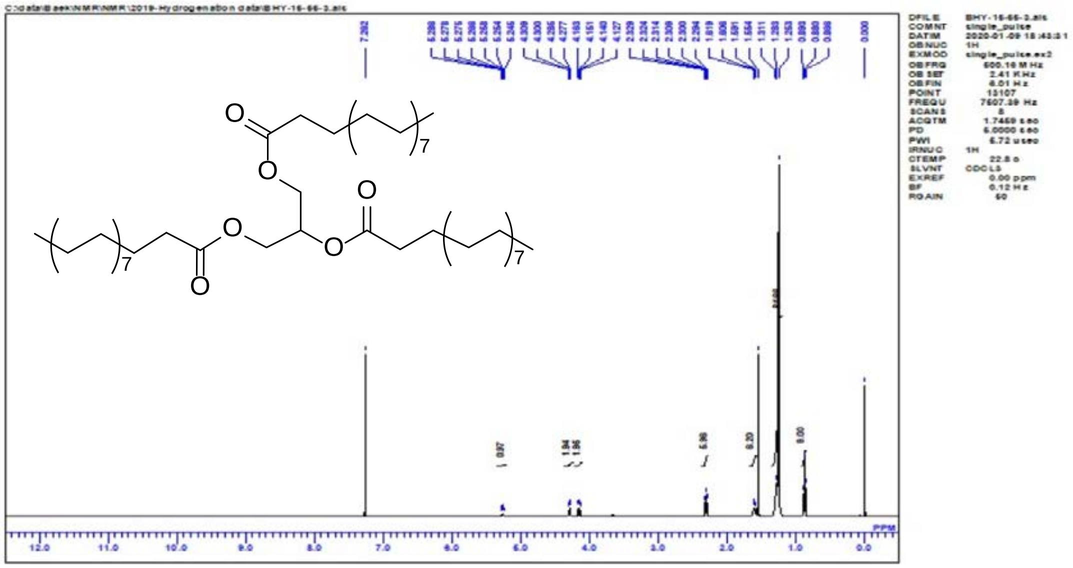

Supplementary Figure 21. ^{1}H NMR Spectra of product **2k**

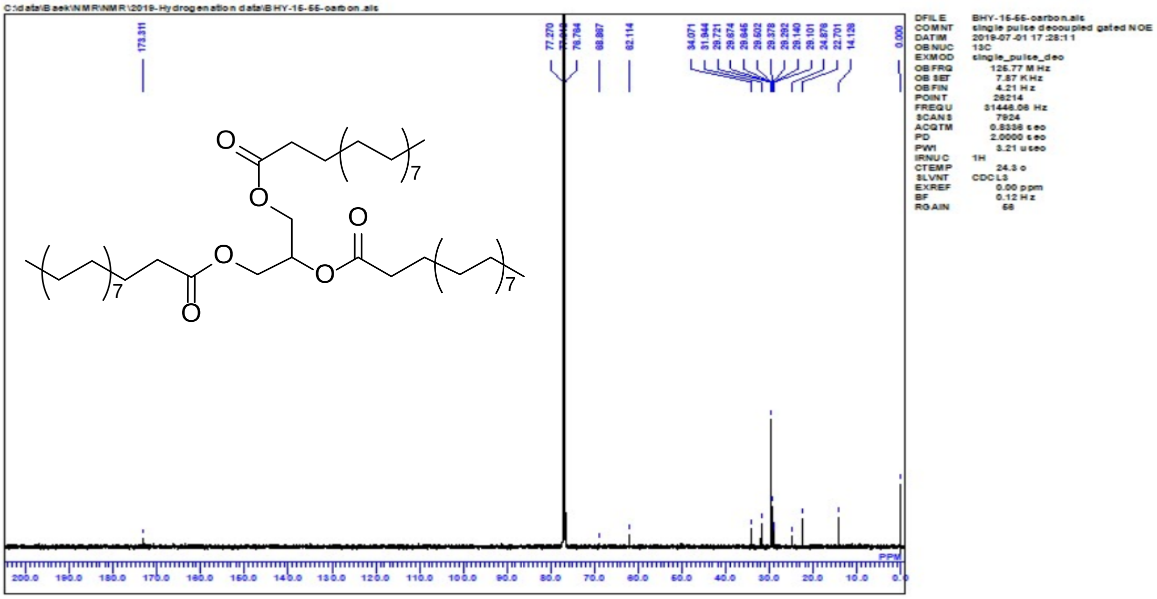

Supplementary Figure 22. ^{13}C NMR Spectra of product **2k**

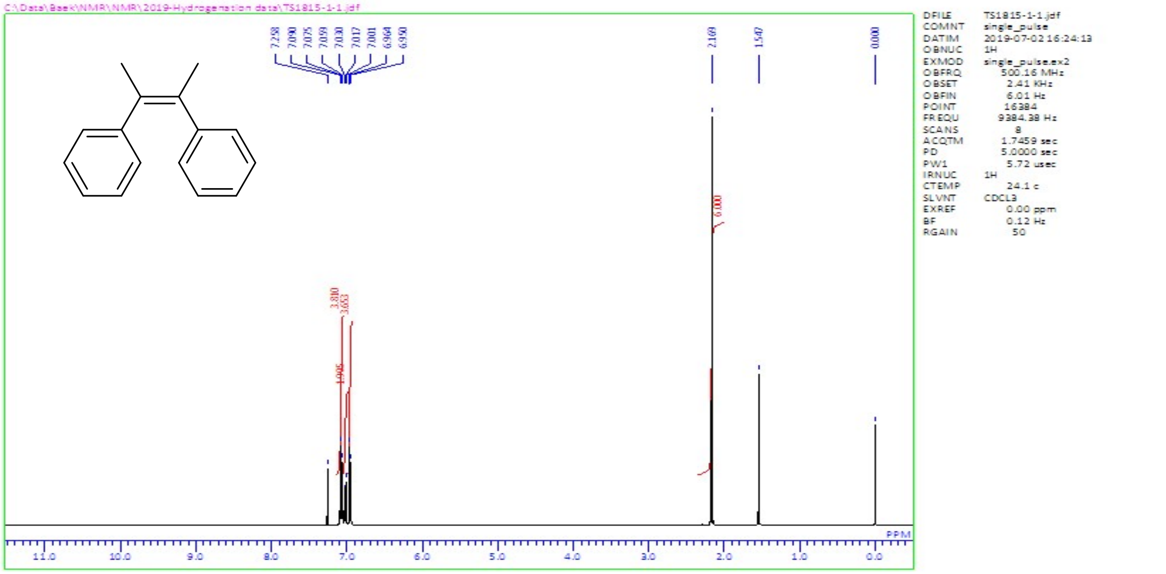

Supplementary Figure 23. ^{1}H NMR Spectra of product **1b**

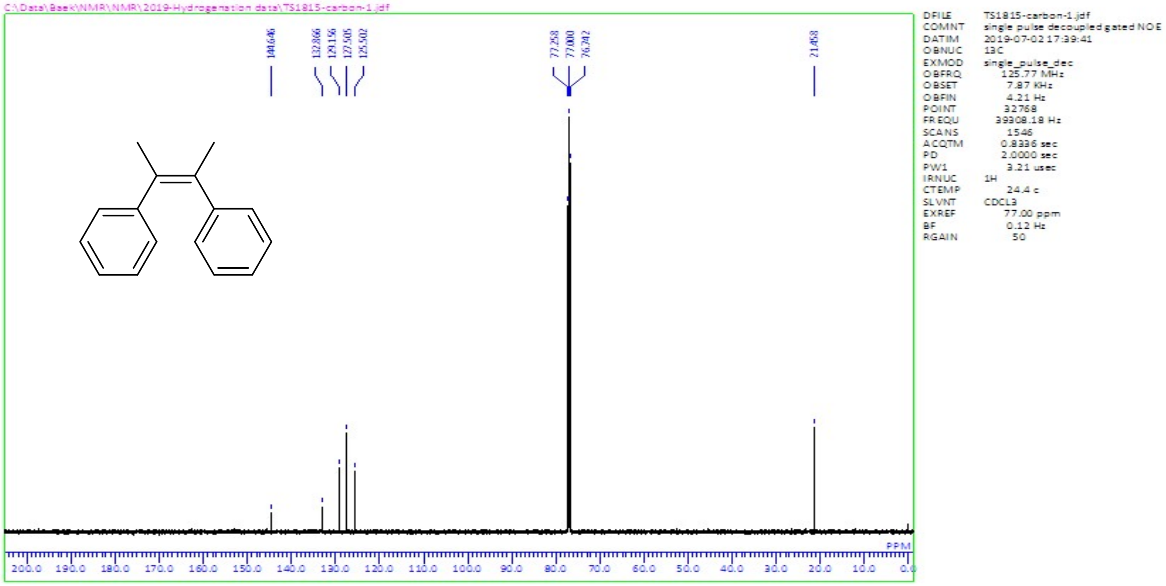

Supplementary Figure 24. ^{13}C NMR Spectra of product **1b**

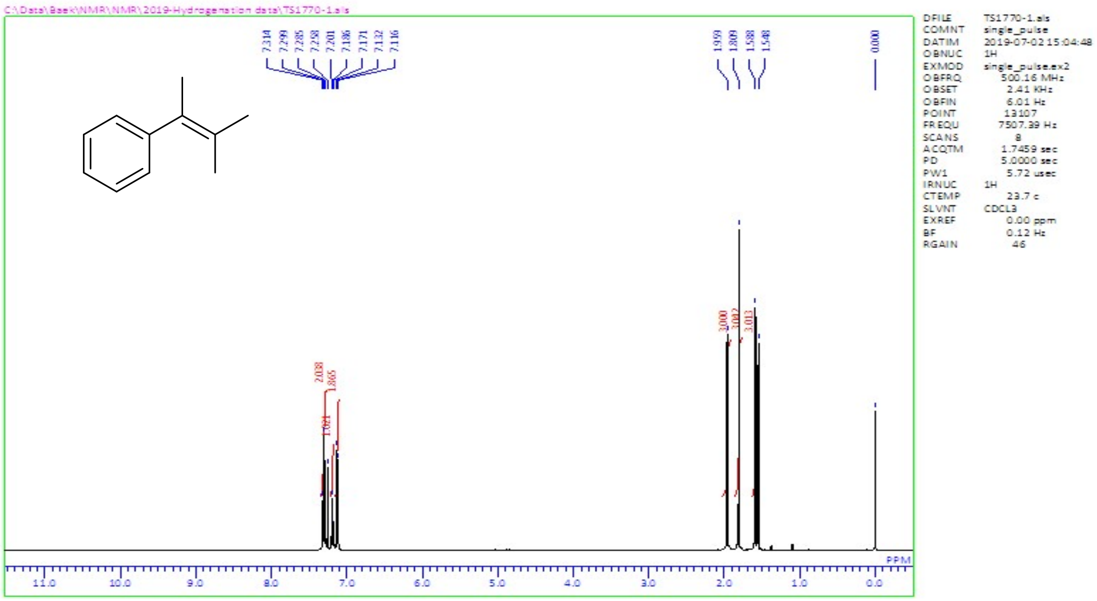

Supplementary Figure 25. ^{1}H NMR Spectra of product **1c**

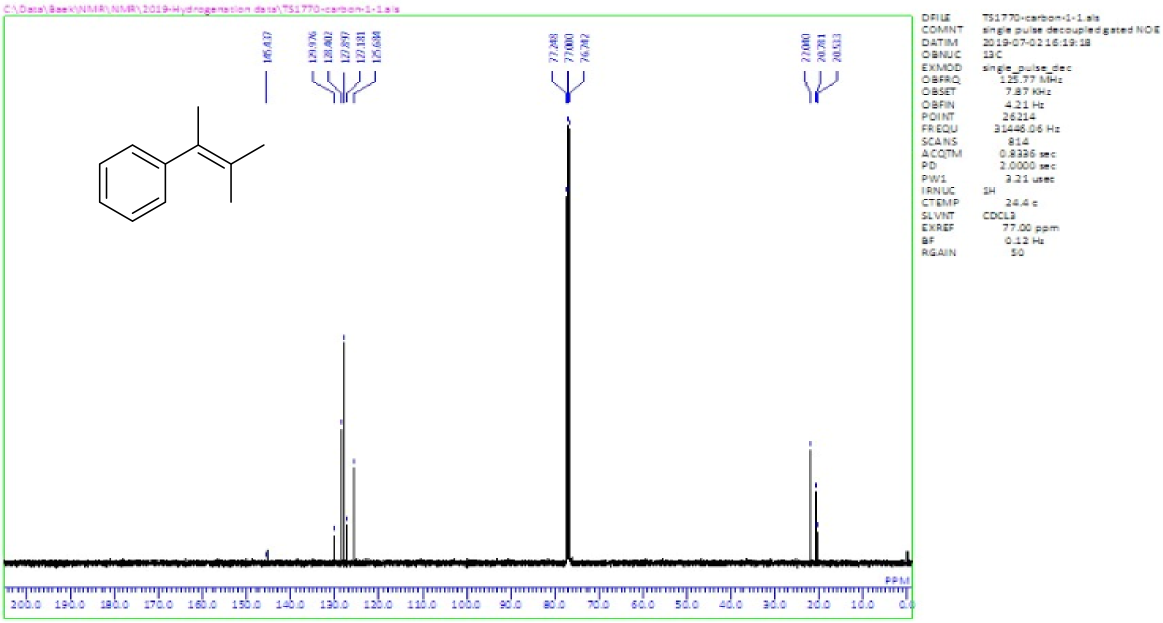

Supplementary Figure 26. ^{13}C NMR Spectra of product **1c**

5. X-ray Structure Analysis

X-Ray Crystal Structure Analysis of 1b.

Crystal data: $C_{16}H_{16}$, FW = 208.29, monoclinic $P2_1/c$, a = 10.2003(4), b = 8.4836(3), c = 14.1475(6) Å, β = 94.953(2)°, V = 1219.69(8) Å^3; D_X = 1.134 Mg m^{-3}; Z = 4; μ(Cu Kα) = 0.475 mm^{-1}, T = 90 K. Block shaped colorless crystals were grown from a CH_2Cl_2/Hexane solution of **1b**. A single crystal with the dimensions of 0.618 x 0.430 x 0.383 mm was mounted on a glass capillary and set on a Rigaku Raxis-Rapid diffractometer. The diffraction data were collected using CuKα radiation, which was monochromated by a graphite monochrometer. The unit cell dimensions were determined using 11625 reflections with 3.135 ≤ θ ≤ 68.270°. The diffraction data of 13457 within 5.141 ≤ θ ≤ 68.234° were collected. The data were merged, including Friedel pairs, to give 2220 unique reflections with the R_{int} of 0.0633. The structure was solved by a direct method and refined on F^2 by a least-squares method by the programs SIR2004[1] and SHELXL-2018/1,[2] respectively. The final R values against 1918 unique reflections (θ_{max} = 68.270°) with $I > 2\sigma(I)$ are 0.0443 and 0.1144 for the $R(F)$ and the $wR(F^2)$, respectively.

The X-ray crystallographic coordinates for structures reported in this Article have been deposited at the Cambridge Crystallographic Data Centre (CCDC), under deposition number CCDC1939498. These data can be obtained free of charge from The Cambridge Crystallographic Data Centre via www.ccdc.cam.ac.uk/data_request/cif.

DOI: 10.5517/ccdc.cc236gx

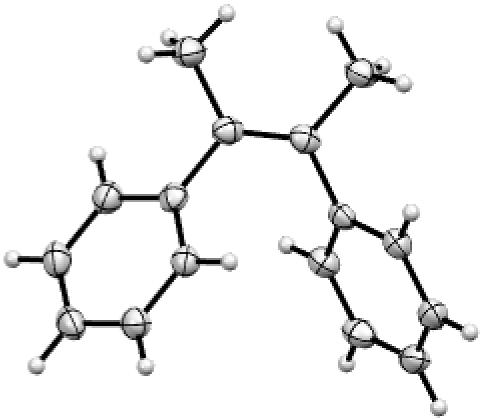

Supplementary Figure 27. ORTEP drawing of compound **1b**.

X-Ray Crystal Structure Analysis of 2b.

Crystal data: $C_{16}H_{18}$, FW = 210.30, monoclinic $P2_1/c$, a = 5.62292(18), b = 17.3124(6), c = 6.9287(2) Å, β = 116.6951(18)°, V = 602.59(3) Å^3; D_X = 1.159 Mg m^{-3}; Z = 2; μ(Cu Kα) = 0.481 mm^{-1}, T = 90 K. Block shaped colorless crystals were grown from a CH_2Cl_2/Hexane solution of **2b**. A single crystal with the dimensions of 0.400 x 0.255 x 0.246 mm was mounted on a glass capillary and set on a Rigaku Raxis-Rapid diffractometer. The diffraction data were collected using CuKα radiation, which was monochromated by a graphite monochrometer. The unit cell dimensions were determined using 4095 reflections with 5.110 ≤ θ ≤ 68.175°. The diffraction data of 6265 within 5.110 ≤ θ ≤ 68.176° were collected and merged to give 1091 unique reflections with the R_{int} of 0.0240. The structure was solved by a direct method and refined on F^2 by a least-squares method by the programs SIR2004[1] and SHELXL-2018/1,[2] respectively. The final R values against

908 unique reflections (θ_{max} = 68.176°) with $I > 2\sigma(I)$ are 0.0436 and 0.1117 for the $R(F)$ and the $wR(F^2)$, respectively.

The X-ray crystallographic coordinates for structures reported in this Article have been deposited at the Cambridge Crystallographic Data Centre (CCDC), under deposition number CCDC 1939526. These data can be obtained free of charge from The Cambridge Crystallographic Data Centre via www.ccdc.cam.ac.uk/data_request/cif.

DOI: 10.5517/ccdc.csd.cc2337cv

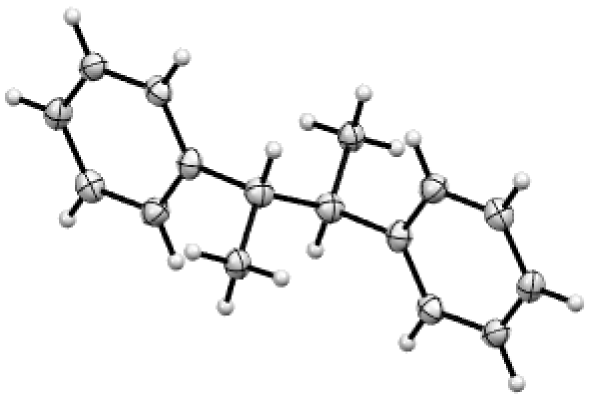

Supplementary Figure 28. ORTEP drawing of compound **2b** (Disordered structure was deleted for clarity).
