# Supplementary material for: Metallically gradated silicon nanowire and palladium nanoparticle composites as robust hydrogenation catalysts
Source: Commun Chem. 2020 Jun 26;3:81. doi: 10.1038/s42004-020-0332-z (PMC9814402; doi:10.1038/s42004-020-0332-z)
Supplement: Supplementary file 2 — Description of Additional Supplementary Files [file 42004_2020_332_MOESM2_ESM.pdf]

Description of Additional Supplementary Files

File Name: Supplementary Data 1
Description: Crystallographic information file for compound **1b**

File Name: Supplementary Data 2
Description: Crystallographic information file for compound **2b**
